# Leveraging Providers’ Preferences to Customize Instructional Content in Information and Communications Technology–Based Training Interventions: Retrospective Analysis of a Mobile Phone–Based Intervention in India

**DOI:** 10.2196/15998

**Published:** 2020-03-03

**Authors:** Hanu Tyagi, Manisha Sabharwal, Nishi Dixit, Arnab Pal, Sarang Deo

**Affiliations:** 1 Carlson School of Management University of Minnesota Minneapolis, MN United States; 2 Max Institute of Healthcare Management Indian School of Business Hyderabad India; 3 Clinton Health Access Initiative New Delhi India

**Keywords:** public health, mobile health, health care providers, health care workers, instructional technology, information technology, infectious diseases, provider training, learning preferences

## Abstract

**Background:**

Many public health programs and interventions across the world increasingly rely on using information and communications technology (ICT) tools to train and sensitize health professionals. However, the effects of such programs on provider knowledge, practice, and patient health outcomes have been inconsistent. One of the reasons for the varied effectiveness of these programs is the low and varying levels of provider engagement, which, in turn, could be because of the form and mode of content used. Tailoring instructional content could improve engagement, but it is expensive and logistically demanding to do so with traditional training

**Objective:**

This study aimed to discover preferences among providers on the form (articles or videos), mode (featuring peers or experts), and length (short or long) of the instructional content; to quantify the extent to which differences in these preferences can explain variation in provider engagement with ICT-based training interventions; and to compare the power of content preferences to explain provider engagement against that of demographic variables.

**Methods:**

We used data from a mobile phone–based intervention focused on improving tuberculosis diagnostic practices among 24,949 private providers from 5 specialties and 1734 cities over 1 year. Engagement time was used as the primary outcome to assess provider engagement. K-means clustering was used to segment providers based on the proportion of engagement time spent on content formats, modes, and lengths to discover their content preferences. The identified clusters were used to predict engagement time using a linear regression model. Subsequently, we compared the accuracy of the cluster-based prediction model with one based on demographic variables of providers (eg, specialty and geographic location).

**Results:**

The average engagement time across all providers was 7.5 min (median 0, IQR 0-1.58). A total of 69.75% (17,401/24,949) of providers did not consume any content. The average engagement time for providers with nonzero engagement time was 24.8 min (median 4.9, IQR 2.2-10.1). We identified 4 clusters of providers with distinct preferences for form, mode, and length of content. These clusters explained a substantially higher proportion of the variation in engagement time compared with demographic variables (32.9% vs 1.0%) and yielded a more accurate prediction for the engagement time (root mean square error: 4.29 vs 5.21 and mean absolute error: 3.30 vs 4.26).

**Conclusions:**

Providers participating in a mobile phone–based digital campaign have inherent preferences for instructional content. Targeting providers based on individual content preferences could result in higher provider engagement as compared to targeting providers based on demographic variables.

## Introduction

The recent proliferation and adoption of information and communications technology (ICT) have the potential to transform learning among health professionals [1]. Riding the technology wave, many public health programs and interventions across geographies and therapeutic areas leverage ICT-based interventions to train and sensitize health professionals [2]. They provide a cost-effective mechanism to reach professionals [3], especially to engage with geographically distant or fragmented providers [4].

However, the evidence regarding the effects of ICT-based interventions on provider knowledge, attitude, practice, and, consequently, patient health outcomes is not unequivocal [5]. One of the reasons for their uncertain effectiveness is low [6] and varying levels [7] of provider engagement within the ICT-based interventions. Among various factors that could explain heterogeneity [8], the format and mode of instructional content are known to play an important role in improving provider engagement with ICT-based interventions [9,10].

Prior research has shown that the customization of instructional content could enhance the learning experience in academic settings among medical students [11]. In the case of nonacademic provider-focused training interventions as well, customization of instructional content could improve provider engagement, but it may not always be feasible. Especially, in the case of traditional training methods such as lectures and conferences, the assessment of learners’ preferences could be expensive, and customized delivery of content could be logistically demanding [12]. In contrast, ICT-based training interventions make it feasible to tailor content to providers’ preferences, providing the much-needed learner-centric approach [13].

This study aimed to determine content preferences among providers in terms of form (articles or videos), mode (featuring peers or experts), and length (short or long) of instructional content. We used the inherent content preferences among providers to explain variation in provider engagement with ICT-based training interventions. We compared the magnitude of variation in provider engagement explained by content preferences with that explained by demographic variables. The research questions have been addressed by analyzing data from a mobile phone–based provider training intervention, which was designed to improve tuberculosis (TB) diagnostic practices among private providers in India.

## Methods

### Study Setting and Participants

We collaborated with a third-party mobile phone–based platform, which helps providers to discuss real-life medical cases with the provider community. At the start of the intervention, more than 225,000 private providers were registered on the platform. For our study, we chose providers who logged into the platform at least once a month and further narrowed our list by choosing providers from 5 specialties—general practice, internal medicine, obstetrics and gynecology, pediatrics, and pulmonology—which account for the bulk of patients with TB initiated on anti-TB treatment [14]. A total of 24,949 private providers spread across 1734 cities and towns in India participated in the campaign, which ran from February to November 2017.

### Intervention

**Figure 1:**
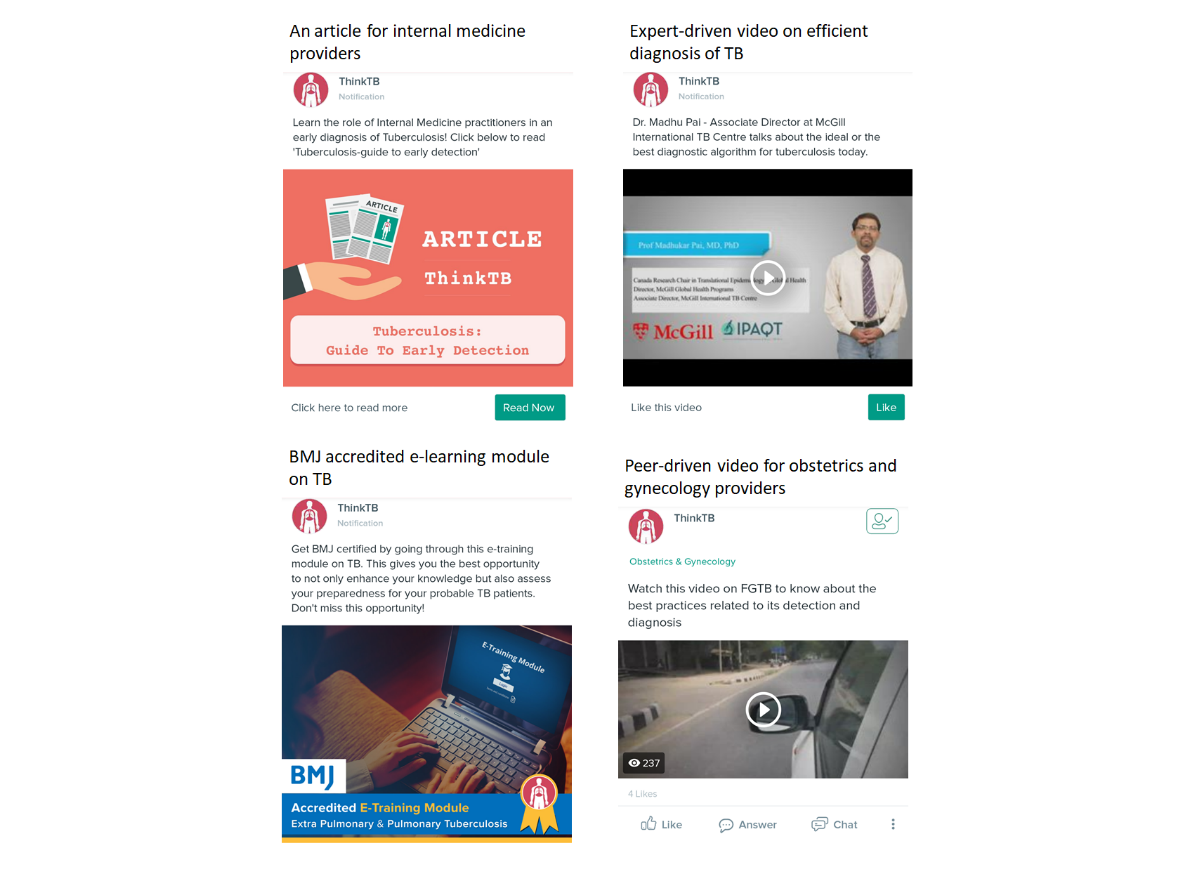
Mobile screenshots of content pieces from the ThinkTB campaign.

We launched a digital campaign on the mobile phone–based platform through a dedicated page called *ThinkTB*. The digital campaign focused on the dissemination of TB diagnostic best practices among private providers in India. It showcased 10 content pieces—5 videos, 3 articles, and 2 interactive games (Table 1). In the first phase, the campaign content aimed at raising awareness and interest among providers. In the second phase, it progressed to interactive content and games to inculcate trial and advocacy. We tailored the content pieces according to the providers’ specialty (Figure 1) to highlight their respective roles in the diagnosis of TB. For example, for video 2 launched in April 2017, the pediatricians were shown a video titled *Role of pediatricians in diagnosing TB*, whereas the gynecologists were shown a video titled *Female genital tract TB: A diagnosing challenge?*

**Table 1:** Details of content pieces delivered in the ThinkTB campaign.

Month and content		Topic	Target specialty
**February 2017**			
	Video 1	Role of GP^a^ in the management of TB^b^ in India Keeping up with the changing diagnostic paradigms in TB Inability to conceive: could this be TB? Role of GP in the management of TB in India	General practice Internal medicine and pulmonology Gynecology General practice
**March 2017**			
	Article 1	Role of GPs in the management of TB in India Vague presentation of TB in pediatric population Inability to conceive: could this be TB? Comparative features of tests for diagnosis of tuberculosis	General practice Pediatrics Obstetrics and gynecology Internal medicine and pulmonology
	Webcast 1	Role of GPs in diagnosis of TB Female genital TB: myths and facts Role of pulmonologists in diagnosing TB Endorsed tests for diagnosis of pulmonary and extra-pulmonary TB	General practice Gynecology Pulmonologists Internal medicine
**April 2017**			
	Video 2	Role of pediatricians in diagnosing TB Female genital tract TB: a diagnosing challenge? Tuberculosis: a growing health concern Tuberculosis: guide to early detection	Pediatrics Gynecology General practice Internal medicine and pulmonology
	Article 2	Female genital tuberculosis: a diagnosing challenge? Tuberculosis: a growing health concern Pediatric tuberculosis: an overview Tuberculosis: all you need to know Tuberculosis: guide to early detection	Gynecology General practice Pediatrics Pulmonology Internal medicine
**June 2017**			
	Article Gyn	An article on drug resistant tuberculosis (10 principles for effective management)	Gynecology
	Calculator	Efficient diagnostic tool	All
**September 2017**			
	Expert video 1^c^	How does one diagnose and treat MDR-TB^d^? How does one treat tuberculosis? How to interpret discordant results? Complex case of tuberculosis Complex case of FGTB^e^ Complex case of drug resistance	All
**October 2017**			
	BMJ training	Accredited E-training module extrapulmonary and pulmonary tuberculosis	All
**November 2017**			
	Expert video 2^c^	What are the recommended tests for pulmonary tuberculosis and which tests are discouraged? What test can be used for diagnosing tuberculosis pleural effusion? What is the ideal or the best diagnostic algorithm for tuberculosis today?	All

^a^GP: general practitioner.

^b^TB: tuberculosis.

^c^The mapping of expert videos with the specialty was 1 to many.

^d^MDR-TB: multidrug-resistant tuberculosis.

^e^FGTB: female genital tuberculosis.

### Data

We had access to 3 datasets pertaining to the providers, content pieces, and the interaction of the provider with the content pieces. The first dataset was unique at a provider level and included provider information—provider specialty and city of practice—which was recorded when the providers registered on the mobile platform. The third-party platform had segmented cities into 3 *city tiers* (tier 1, tier 2, and tier 3) based on a classification used by the Indian government to set the minimum daily wage for agricultural and industrial workers in India and estimate the house rent allowance provided by the Central Government of India to its employees [15]. The second dataset was unique at the content piece level and included classification of each content piece by its format (articles or videos), mode (videos featuring either peers or subject matter experts from the provider community), and length (long content, which is >10 min, or short content, which is ≤10 min) as shown in Table 2. The third dataset was unique at the provider-content piece level and recorded the engagement time defined as the time spent by a provider on a content piece (reading or viewing). We defined the content piece to be *consumed* if the engagement time associated with the content piece was greater than zero.

We combined the 3 datasets to obtain a comprehensive dataset with 24,949 observations, each representing a unique provider. It contained the following variables: provider specialty; city; city tier; the engagement time spent on each content piece; and the format, mode, and length of the content pieces. We excluded providers who had zero engagement time and further removed outliers with engagement time greater than the sum of the third quartile and 1.5 times the IQR. The remaining providers with nonzero engagement time were taken into consideration for analysis.

**Table 2:** Categorization of content pieces.

Content piece	Format	Length	Mode^a^
Article 1	Article	Short	N/A^b^
Video 1	Video	Short	Peer
Webcast 1	Video	Long	Peer
Article 2	Article	Short	N/A
Video 2	Video	Short	Peer
Calculator	Article	Short	N/A
Article Gyn	Article	Short	N/A
Expert video 1	Video	Short	Expert
Expert video 2	Video	Short	Expert
BMJ training	Article	Long	N/A

^a^Mode is only defined for videos.

^b^Not applicable.

### Analysis

We used engagement time with the campaign as the primary outcome to assess provider engagement. We conducted our analysis in 2 steps as has been described in the following subsections. As described in the subsection *Discovering Content Preferences Among Providers*, providers were segmented into clusters based on the proportion of time spent on content formats and modes to discover their content preferences. As explained in the subsection *Predicting Provider Engagement*, separate regression models were fitted to assess the extent to which the variation in engagement time can be explained by clusters and demographic variables. All analyses were performed using R 3.4.3 (The R Foundation) [16].

#### Discovering Content Preferences Among Providers

We calculated 3 proportions of engagement time spent by a provider using the content classifications described earlier. For the 2 formats (text and video), *read proportion* was calculated as the proportion of engagement time spent by a provider on reading articles. For the 2 modes of videos (peer and expert), *expert proportion* was calculated as the proportion of engagement time spent by a provider on videos featuring an expert. For the 2 types of lengths, *short proportion* was calculated as the proportion of engagement time spent on consuming short content pieces.

These 3 proportions were used to cluster providers using the k-means algorithm [17]. The k-means algorithm partitioned providers into clusters such that the providers were well matched to other providers in their own clusters but were very different from those in the other clusters. The optimal number of clusters was determined using the average silhouette width method [18]. A higher value of silhouette width of observation indicates that the observation is well matched to its cluster and poorly matched to neighboring clusters. We varied the number of clusters from 2 to 15 to determine an optimal number of clusters. Moreover, Chi-square tests were performed to assess the similarity of clusters in terms of the city tier and specialty distribution among them.

#### Predicting Provider Engagement

We estimated 2 linear regression models to explain the variation in provider engagement with the campaign using engagement time as the outcome variable. For the first model (model A), clusters were used as a predictor variable. For the second model (model B), provider-level demographic variables (specialty and city tier) were used as predictor variables. The number of providers in the regression models was 6482 after data cleaning, as has been explained in previous sections.

The demographic variable–based model was compared with the cluster-based model based on its predictive power. A 10-fold cross-validation method was used, which is commonly used for model selection [19]. For each dataset, 9 parts were used for training the model and the tenth part for testing it. This process was repeated 10 times, ensuring that each dataset partition served as a test set. We then compared the average of cross-validation root mean square error (RMSE) and mean absolute error (MAE) for the 2 models.

## Results

### Dataset

**Figure 2:**
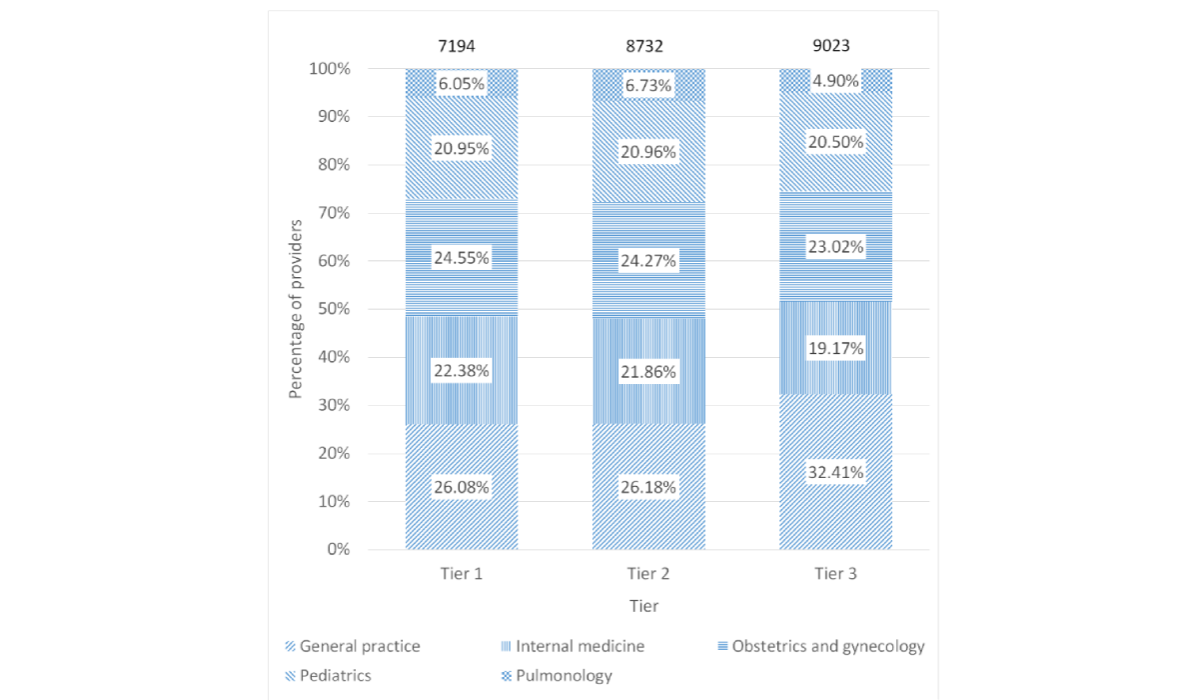
Provider count in the activity dataset by specialty and city tier.

The comprehensive dataset consisted of 24,949 providers. Table 3 shows the distribution of these providers by specialty and city tier. On one hand, general practice physicians accounted for the largest share of providers by specialty. On the other hand, tier 3 cities accounted for the largest share of providers by city tiers. As shown in Figure 2, the mix of providers by specialty varied across the 3 city tiers (Chi-square*P*P<). The median engagement time for 24,949 providers was 0 min (mean 7.5, IQR 0-1.58), as 69.75% (17,401/24,949) of providers did not consume any content. Moreover, 30.25% (7548/24,949) of providers consumed at least one content piece. The engagement among providers with engagement time greater than 0 min varied significantly, as the median engagement time was 4.9 min (mean 24.8, IQR 2.2-10.1).

We excluded 69.75% (17,401/24,949) providers who had zero engagement time and further removed 1.36% (339/24,949) outliers with engagement time greater than the sum of the third quartile and 1.5 times the IQR. As described in the Analysis section, we calculated read proportion, expert proportion, and short proportion for providers. We excluded 2.91% (727/24,949) providers who had undefined proportions (zero divided by zero) for the calculated proportions. Our final dataset contained 6482 providers after removing outliers. The engagement time for these providers, too, varied with median engagement time of 5.2 min (mean 6.6, IQR 2.42-9.83). It also varied by provider specialty and city tier. Among provider specialty, general practice physicians recorded the highest average engagement of 7.2 min, whereas internal medicine physicians recorded the lowest at 6.09 min. Among city tiers, tier 3 providers engaged the highest with the platform, spending an average of 6.8 min, whereas tier 1 providers engaged the lowest at 6.3 min. We conducted one-way analysis of variance tests and found that the engagement time was statistically different across specialty (*P*<.001) and city tiers (*P*=.007).

**Table 3:** Provider participation by city tier and specialty.

City tiers	Specialty, n					
	General practice	Internal medicine	Obstetrics and gynecology	Pediatrics	Pulmonology	Total
Tier 1	1876	1610	1766	1507	435	7194
Tier 2	2286	1909	2119	1830	588	8732
Tier 3	2924	1730	2077	1850	442	9023
Total	7086	5249	5962	5187	1465	24,949

### Discovering Content Preferences Among Providers

**Figure 3:**
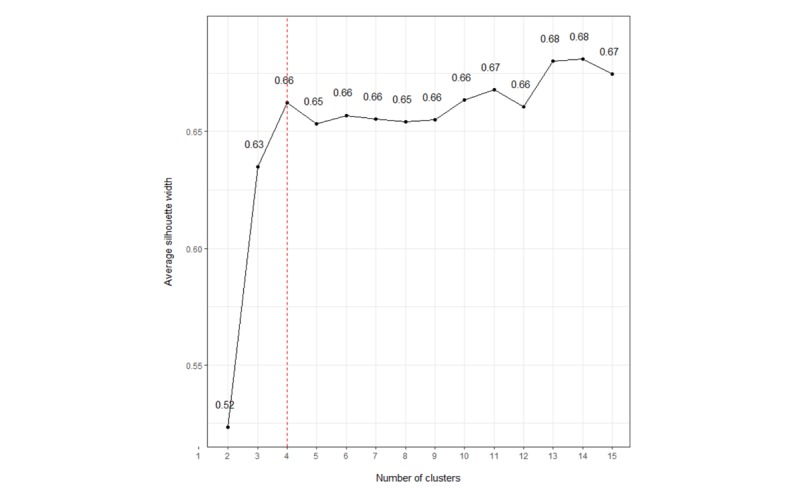
Optimal number of clusters using the average silhouette width method.

The average silhouette width was the highest at 0.68 for 14 clusters (Figure 3). However, we chose the number of clusters to be 4, with a marginally lower average silhouette width of 0.66 because of the ease of interpretability of the resulting clusters. Table 4 describes the clusters and their characteristics, which has been explained as follows.

**Table 4:** Clusters and their characteristics.

Cluster	Providers, n	Engagement time spent reading, %	Engagement time spent on short content, %	Engagement time spent on expert-driven content, %	Average silhouette width
1. Peer-driven microwatchers	2425	1.7	97.3	2.0	0.81
2. Expert-driven microwatchers	772	5.7	98.8	92.7	0.75
3. Peer-driven microreaders	923	50.7	87.5	3.1	0.34
4. Peer-driven long watchers	2362	5.9	20.5	2.3	0.61
Total	6482	10.7	68.1	13.1	0.66

The largest cluster containing 2425 providers was labeled as *peer-driven microwatchers.* It was also the cluster with the highest average silhouette width (0.81), indicating that there was higher homogeneity within the cluster when compared with heterogeneity across other clusters. Providers in this cluster spent 98.27% (8493/8642 min) of their time watching videos. They spent 97.28% (8408/8642 min) of their time engaging with short content. Between video modes, they preferred peer-driven content, as they spent 97.95% (8097/8266 min) of their time on peer-driven content.

The second cluster was labeled as e*xpert-driven microwatchers*, which was the smallest cluster with 772 providers. Providers in this cluster spent 94.27% (2850/3024 min) of their time watching videos and 98.85% (2989/3024 min) of their time engaging with short content. However, in contrast with providers in the first cluster, providers in the second cluster spent 92.67% (2526/2726 min) of their time watching expert-driven videos, indicating that they preferred expert-driven content over peer-driven content.

The third cluster was labeled as *peer-driven microreaders*. This cluster had 923 providers and registered the smallest average silhouette width of 0.34, indicating that the providers in this cluster were less similar among themselves than providers in other clusters. Providers in this cluster spent more time reading (50.65%, 3870/7640 min) than watching (49.35%, 3770/7640 min). They preferred short content and peer-driven videos, as they spent 87.52% (6687/7640 min) on short content and 96.95% (3924/4047 min) of their time on peer-driven videos.

The fourth cluster with 2362 providers was labeled as *peer-driven long watchers*. Providers in this cluster spent 94.06% (22,288/23,696) of their time watching videos and 97.70% (21,535/22,041 min) of their time watching videos that featured peers. Moreover, they spent 79.51% (18,841/23,696 min) of their time engaging with longer content.

**Figure 4:**
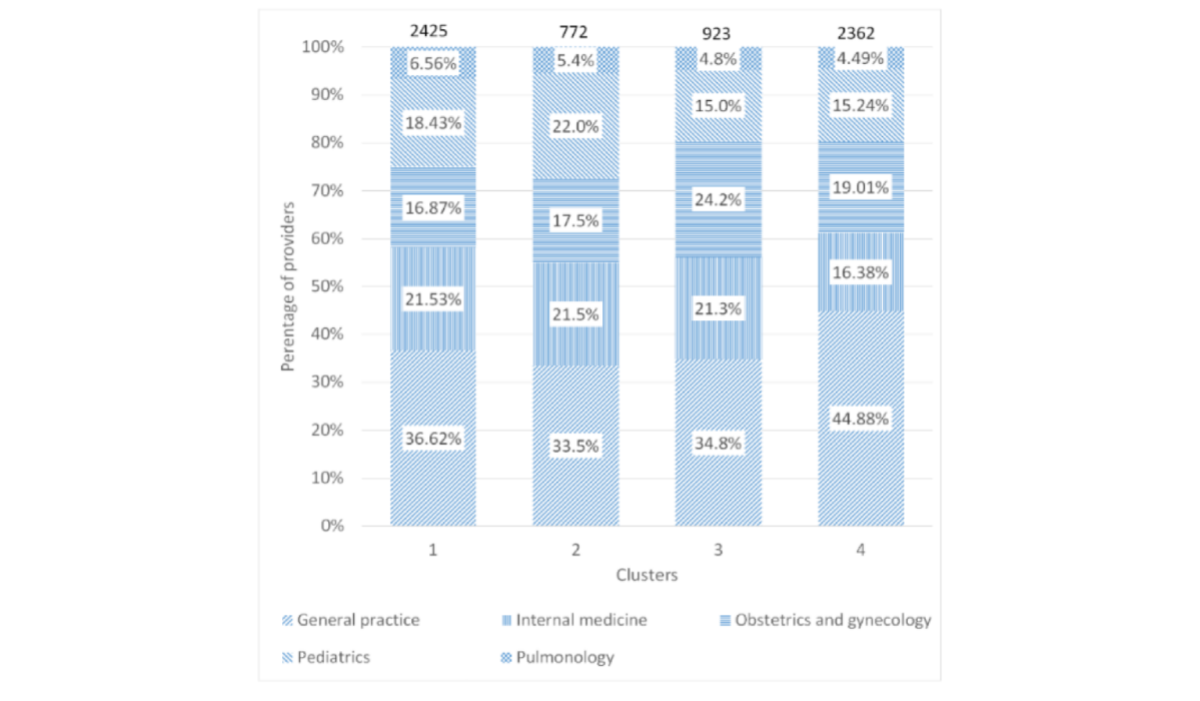
Composition of clusters by specialty.

**Figure 5:**
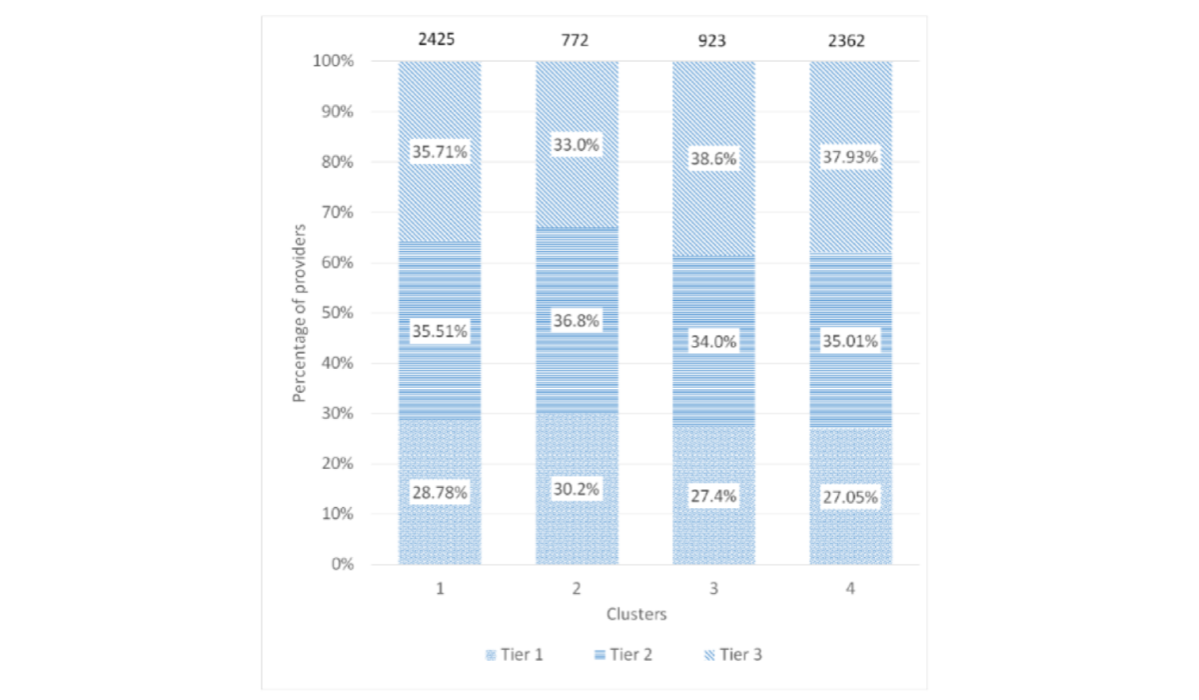
Composition of clusters by city tier.

Figures 4 and 5 show the composition of clusters by specialty and city tier, respectively. The proportion of general practice physicians was between 33.5% (259/772) and 44.88% (1060/2362), whereas the proportion of tier 1 providers was between 27.05% (639/2362) and 30.2% (233/772) across all 4 clusters. The Chi-square tests revealed that the city tier mix was similar across the clusters (*P*=.17), but the specialty mix among the clusters was different (*P*<.001).

### Predicting Provider Engagement

Table 5 shows the results from the linear regression models that use clusters (model A) and demographic variables (model B) to explain variation in provider engagement. For model A, the *R^2^* statistic was 0.329, whereas the *R^2^* statistic for model B was 0.010. In other words, clusters were able to explain a significantly higher proportion of the variation in engagement time when compared with that explained by demographic variables (32.9% vs 1.0%).

**Table 5:** Regression results for model A (cluster-based model) and model B (demographic variable–based model).

Independent variables			*Dependent variable* (engagement time)		
			Model A- Coefficient (standard error)^a,b^		Model B- Coefficient (standard error)^a,c^
Cluster 2			0.353^d^ (0.177)		N/A^e^
Cluster 3			4.713^f^ (0.166)		N/A
Cluster 4			6.468^f^ (0.124)		N/A
Specialty, internal medicine			N/A		−1.116^f^ (0.179)
Specialty, obstetrics and gynecology			N/A		−1.030^f^ (0.182)
Specialty, pediatrics			N/A		−0.644^f^ (0.187)
Specialty, pulmonology			N/A		−0.902^f^ (0.297)
City tier, tier 2			N/A		0.364^d^ (0.164)
City tier, tier 3			N/A		0.393^d^ (0.162)
Constant			3.564^f^ (0.087)		6.934^f^ (0.147)

^a^Observations used: 6482

^b^*R^2^*: 0.329; Adjusted *R^2^*: .0329

^c^*R^2^*: 0.010; Adjusted *R^2^*: .0.009

^d^*P*<.05.

^e^Not applicable.

^f^*P*<.01.

Table 6 compares the RMSE, *R^2^* statistic, and MAE from the 2 predictive models based on the 10-fold cross-validation method. In addition to being able to explain a significantly higher proportion of variation in engagement time, we observed that model A also resulted in 17.7% lower RMSE and 22.7% lower MAE than that of model B.

**Table 6:** Comparison between regression models for engagement time based on 10-fold cross-validation error rates.

Evaluation metrics	Model A based on behavioral variables (clusters)	Model B based on demographic variables (specialty, city tier)	Difference (%; calculated as model B −model A)/model B)
Root mean square error	4.29	5.21	17.7
*R*^2^ statistic	0.33	0.01	−3275.7
Mean absolute error	3.30	4.26	22.7

## Discussion

### Principal Findings

This study analyzed a mobile phone–based campaign that focused on educating private providers in India on TB diagnostic practices. It was found that there is heterogeneity in provider engagement with ICT-based training interventions. We also found that providers have inherent preferences for the type of instructional content. Content preferences were used to cluster providers, and it was shown that these clusters explain the variation in provider engagement better than the demographic variables such as provider specialty and city tier.

ICT-based public health interventions are often low intensity, have a wide reach, and witness low provider engagement [20]. In our study, providers spent an average of 7.5 min with the yearlong ThinkTB campaign, which is lower than that observed in similar interventions across the world [21]. However, the relatively lower engagement may be because of a significantly wider reach when compared with similar precedents, indicating an engagement vs reach trade-off in provider training interventions. Similar to previous studies, provider engagement with the ThinkTB campaign varied significantly, which could have affected the overall effectiveness of the campaign. The variation in provider engagement is important because there is some evidence that provider engagement and effectiveness of public health interventions are closely related [22]. In particular, providers who have lower engagement with the intervention often have worse patient outcomes [21,23].

#### Discovering Content Preferences Among Providers

Our clustering analysis identified groups of providers with homogeneous content preferences despite inherent individual content preferences among providers. Notably, it did not feature all possible combinations of content format, mode, and length. For example, there is no cluster of providers who prefer to watch longer videos featuring experts. This is because, given the number of clusters, the k-means algorithm chooses clusters with the highest average silhouette width across clusters. An increase in the number of clusters would mean poorer interpretability, and thereby, lower feasibility of catering to those additional clusters.

Our clustering analysis confirmed prior research, which shows that health care professionals are known to have individual content preferences [11]. One of the methods used to study instructional content preferences among medical students is the VARK model, which measures preferences for 4 content forms—visual, auditory, reading/writing, and kinesthetic (VARK) [23]. Our study differed from such studies in two ways. First, prior work studied content preferences in an academic context, largely to educate medical students. In contrast, we studied content preferences among practicing providers through a digital campaign geared toward changing their diagnostic behavior. Second, our study identified preferences for unexplored content types that are important for ICT-based interventions to engage with practicing providers in a nonacademic setting. For example, our campaign included 2 of the 4 content forms proposed by the VARK model—visual (videos) and reading/writing (articles). In addition to the content format (video or article), our study also identified preferences for other content types—mode (expert-driven or peer-driven) and length (long or short)—which could provide insights for designing future ICT-based interventions.

Our clustering analysis highlighted two themes that were common for most of the providers. First, providers, on average, preferred shorter content. This is an intuitive outcome, especially for Indian health professionals who experience high burnout rates and work-related stress [24,25]. Given the time constraints, they would presumably prefer to consume shorter content on ICT-based platforms. Second, most providers, on average, preferred watching videos to reading articles. The VARK model suggests that instructional content preferences among medical students vary by study. In particular, there is no clear preference between video and reading/writing content forms among medical students [26-28]. However, our results showed that, on average, providers have a strong preference for video content, as they spent only 10.7% of their time reading articles (Table 4). This difference in results could be because we studied content preferences among practicing providers in a nonacademic setting, unlike studies involving VARK models, which studied content preferences among medical students in an academic setting.

Although the literature is mostly equivocal on the impact of catering to such preferences [29], there is some evidence that customization could result in efficient and effective learning [30]. Within health care settings as well, catering to individual preferences of medical practitioners participating in health care interventions is known to drive the effectiveness of health care interventions [31]. Although content preferences may be individual to every provider, our clustering analysis shows that they could be identified at a group level too.

#### Predicting Provider Engagement

A comparison of prediction models revealed that clusters based on content preferences predicted engagement time better than demographic variables. We also showed that the composition of clusters varied by specialty but not by city tier, thereby implying that demographic variables may not always be associated with content preferences, and hence, the ability of demographic variables to explain the variation in behavior may be different from that of clusters formed on the basis of content preferences.

At first glance, better prediction of engagement using clusters may seem obvious because clusters were created using a measure of engagement time. However, it is important to note that we used the *proportion of engagement time* spent by providers on various types of content and not the absolute magnitude of the engagement time. This implies that 1 cluster can contain providers with varying levels of engagement (low and high) as long as the proportions of time spent on different content types are similar. Hence, clusters are not guaranteed to provide a better prediction of engagement time by definition.

### Recommendations

Using content preferences to engage with providers has important implications for provider-focused training interventions. Provider-focused training interventions often target providers based on demographic variables, such as geographic location (rural, urban, etc), provider specialty (internal medicine, family practice, etc), or clinical setting (university, private practices, etc) [32-34]. Such interventions are unable to recognize and leverage heterogeneity in content preferences at an individual level. Our study provides evidence that suggests moving away from provider demographic information. We propose that training interventions leverage ICT-based platforms to learn individual preferences and deliver customized content based on provider preferences instead of demographic variables.

Learning provider preferences could be operationally challenging, but ICT tools could act as the enabler of the proposed approach. When compared with traditional counterparts, such as physical outreach visits and conferences, ICT-based training interventions are flexible in terms of training timing and sequences and improved access to geographically dispersed providers [35]. Their interactive nature allows dynamic assessment of learners’ preferences without which public health interventions resort to using demographic variables for engaging with providers. In addition, ICT-based interventions are adaptable to deliver customized content based on individual content preferences [36]. Therefore, it is feasible to customize instructional content for public health interventions because of ICT-based solutions.

### Limitations

Our study has certain limitations. Providers may have left the mobile screen open for a prolonged period without actually engaging with the campaign. Hence, the engagement time recorded on the platform might not reflect the actual time spent by the provider. We partially addressed this issue by removing outliers from our analyses.

Our study offers limited generalizability because of three reasons. First, our clustering analysis and prediction models excluded 69.75% (17,401/24,949) of providers, who had zero engagement time with the campaign. We could not assess the preferences of providers who did not engage with the campaign, which may limit the generalizability of findings to providers who did not engage with the content. A large proportion of unresponsive providers may have also introduced a selection bias. It is possible that providers who did not engage with the campaign did not find ThinkTB relevant or did not engage with the mobile platform at all. However, this did not affect our insights into content preferences, as the heterogeneity in engagement time of providers who had nonzero engagement time allowed us to perform clustering.

Second, the fact that provider clusters in our study were based on individual content preferences limits the generalizability of our results. The content preferences are unique to a provider, which limits the generalizability of our results to other contexts such as another training subject with different content types delivered via a technology platform that is not mobile phone based. Nonetheless, the identification of unique preferences and catering to those preferences is generalizable. On the basis of our results, engaging with providers based on their individual preferences instead of demographic variables could lead to higher engagement with the intervention.

Third, the campaign focused on educating private providers in India on TB diagnostic practices. Providers on the mobile phone–based platform either may not be interested in this topic or may not identify with the platform for this topic. Hence, results from this study may not be generalizable to other clinical contexts other than TB. However, the clinical context of TB in itself is significant in scope. TB is the leading cause of death from a single infectious agent, ranking above HIV/AIDS [37]. Eradication of TB therefore has been a global priority. The World Health Organization (WHO) designed the End TB Strategy in 2014 and subsequently, the United Nations General Assembly included *ending the TB epidemic* as one of the Sustainable Development Goals in 2015 [38]. Moreover, India—the geographical focus of our study—contributes more than a fourth of global TB incidence [37]. The private sector in India is estimated to account for half of the patients with TB in India [39] and is known to be suboptimal in their TB diagnostic and treatment practices [40]. Private sector engagement models in India are being scaled to multiple cities [41], but these physical engagement models are resource intensive [42]. Our campaign was one of the first ICT-based interventions that could provide a more sustainable option of engaging with private providers. Beyond India, too, engaging with private providers is identified as a global priority. WHO asserted that engaging with the private sector could account for 3.6 million missing TB cases globally and proposed the adoption of the Public-Private Mix model to improve TB detection and treatment [43]. Therefore, our study has implications for interventions aimed at engaging with private providers for TB care across the globe.

### Conclusions

Our study shows that providers participating in a mobile phone–based digital campaign have inherent preferences for the instructional content. It also shows that targeting providers by catering to individual provider content preferences could result in a higher provider engagement when compared with targeting them based on demographic variables. A higher provider engagement could maximize provider learning and improve the effectiveness of public health interventions. ICT allows us to cater to individual content preferences and could be leveraged to design provider-centric health interventions.

## Abbreviations

ICT: information and communications technology
MAE: mean absolute error
RMSE: root mean square error
TB: tuberculosis
VARK: visual, auditory, reading/writing, and kinesthetic
WHO: World Health Organization
